# Sentinel node involvement with or without completion axillary lymph node dissection: treatment and pathologic results of randomized SERC trial

**DOI:** 10.1038/s41523-021-00336-3

**Published:** 2021-10-08

**Authors:** Gilles Houvenaeghel, Monique Cohen, Pédro Raro, Jérémy De Troyer, Pierre Gimbergues, Christine Tunon de Lara, Vivien Ceccato, Véronique Vaini-Cowen, Christelle Faure-Virelizier, Frédéric Marchal, Tristan Gauthier, Eva Jouve, Pierrick Theret, Claudia Regis, Philippe Gabelle, Julia Pernaut, Francesco Del Piano, Gauthier D’Halluin, Stéphane Lantheaume, Emile Darai, Bassoodéo Beedassy, Caroline Dhainaut-Speyer, Xavier Martin, Sophie Girard, Richard Villet, Emilie Monrigal, Théophile Hoyek, Jean-François Le Brun, Pierre-Emmanuel Colombo, Agnès Tallet, Jean-Marie Boher, SERC trial group

**Affiliations:** 1 Institut Paoli Calmettes & CRCM & Aix Marseille Univ, 232 Bd de Sainte Marguerite, 13009 Marseille, France; 2 Department of Surgery, Institut Paoli Calmettes & CRCM & Aix Marseille Univ, 232 Bd Ste Marguerite, Marseille, France; 3 Institut de Cancérologie de l’Ouest - Site Paul Papin, 5 rue André Boquel, 10059 49055 Angers Cedex 02 CS, France; 4 Polyclinique Urbain V, Chemin du Pont des Deux Eaux, 84000 Avignon, France; 5 Centre Jean Perrin, 58 rue Montalembert BP 392, 63011 Clermont Ferrand Cedex, France; 6 Institut Bergonie, 229 cours de l’Argonne, 33076 Bordeaux Cedex, France; 7 Institut Jean Godinot, 1 rue du Général Koenig, 51056 Reims, France; 8 Clinique du Parc Rambot 2, Avenue du Dr Aurientis, 13100 Aix en Provence, France; 9 Centre Léon Bérard, 28 rue Laennec, 69373 Lyon Cedex 8, France; 10 Centre Alexis Vautrin, 6 avenue de Bourgogne, 54511 Vandoeuvre-lès-Nancy, France; 11 CHU Dupuytren, 2 avenue Martin Luther King, 87000 Limoges, France; 12 Institut Universitaire du Cancer Toulouse, Oncopole, 1 avenue Irène Joliot-Curie, 31059 Toulouse, France; 13 CH Saint Quentin, 1 avenue Michel de l’Hospital, B.P. 608, 02321 Saint Quentin Cedex, France; 14 Centre Oscar Lambret, 3 rue F. Combemal, 59000 Lille, France; 15 GHM de Grenoble, La Clinique des Eaux Claires, 8 rue du Dr Calmette, 38028 Grenoble Cedex 1, France; 16 Groupe Santé Victor Pauchet, 61 rue Alexandre Dumas, 80090 Amiens, France; 17 Hôpitaux Du Léman, 3 avenue de la Dame, 74200 Thonon, France; 18 Centre Clinical, 2 chemin Frégenueil CS 42510 Soyaux, 16025 Angoulème, France; 19 Clinique Pasteur, 294 boulevard Charles de Gaulle, 07500 Guilherand Granges, France; 20 Hôpital Tenon, 4 rue de la Chine, 75020 Paris, France; 21 Hôpital Sainte Musse (CHITS), Service de chirurgie viscérale, Rue Henri Sainte-Claire Deville, 83056 Toulon, France; 22 GCS Recherche et Innovation Sante Sarcelles, 6 avenue Charles Péguy, 95200 Sarcelles, France; 23 Hôpital Prive Sainte Marie, 4 allée Saint Jean les Vignes, 71100 Chalon sur Saône, France; 24 Centre Hospitalier Alpes Léman, 558 Route de Findrol BP 20500, 74130 Contamine sur Arve, France; 25 Groupe Hospitalier Des Diaconesses Croix Saint Simon, Site Reuilly, 18 rue Sergent Bauchat, 75012 Paris, France; 26 Clinique Clémentville, 25 rue de Clémentville, 34070 Montpellier, France; 27 Centre Hospitalier Auxerre, 2 bd de Verdun, BP 69, 89011 Auxerre, France; 28 Centre Francois Baclesse, Avenue du Général Harris, 14076 Caen Cedex 5, France; 29 ICM – Institut Régional du Cancer Montpellier, 208 avenue des Apothicaires – Parc Euromédecine, 34298 Montpellier Cedex 5, France; 30 Department of Radiotherapy, Institut Paoli Calmettes & CRCM & Aix Marseille Univ, 232 Bd Ste Marguerite, Marseille, France; 31 Aix Marseille Univ, INSERM, IRD, SESSTIM, Marseille, France

**Keywords:** Cancer, Surgical oncology

## Abstract

Based on results of clinical trials, completion ALND (cALND) is frequently not performed for patients with breast conservation therapy and one or two involved sentinel nodes (SN) by micro- or macro-metastases. However, there were limitations despite a conclusion of non-inferiority for cALND omission. No trial had included patients with SN macro-metastases and total mastectomy or with >2 SN macro-metastases. The aim of the study was too analyze treatment delivered and pathologic results of patients included in SERC trial. SERC trial is a multicenter randomized non-inferiority phase-3 trial comparing no cALND with cALND in cT0-1-2, cN0 patients with SN ITC (isolated tumor cells) or micro-metastases or macro-metastases, mastectomy or breast conservative surgery. We randomized 1855 patients, 929 to receive cALND and 926 SLNB alone. No significant differences in patient’s and tumor characteristics, type of surgery, and adjuvant chemotherapy (AC) were observed between the two arms. Rates of involved SN nodes by ITC, micro-metastases, and macro-metastases were 5.91%, 28.12%, and 65.97%, respectively, without significant difference between two arms for all criteria. In multivariate analysis, two factors were associated with higher positive non-SN rate: no AC versus AC administered after ALND (OR = 3.32, *p* < 0.0001) and >2 involved SN versus ≤2 (OR = 3.45, *p* = 0.0258). Crude rates of positive NSN were 17.62% (74/420) and 26.45% (73/276) for patient’s eligible and non-eligible to ACOSOG-Z0011 trial. No significant differences in patient’s and tumor characteristics and treatment delivered were observed between the two arms. Higher positive-NSN rate was observed for patients with AC performed after ALND (17.65% for SN micro-metastases, 35.22% for SN macro-metastases) in comparison with AC administered before ALND.

## Introduction

The most commonly accepted prognostic factors for proposing adjuvant systemic therapy in breast cancer (BC) include patient age, tumor size, axillary lymph node status, tumor pathology including grade, lymphovascular invasion (LVI), endocrine receptor (ER) status, Her2 status, and proliferation assays such as the Ki67 labeling index^1–5^. Axillary lymph node involvement remains a major prognostic factor.

Sentinel lymph node biopsy (SLNB) is the recommended surgical procedure for patients with BC with clinically N0 status based on results of randomized studies^6,7^ without completion axillary lymph node dissection (cALND).

Axillary lymph node involvement rate in early BC is about 35–36%, with 3% pN0(i+), 8% pN1mi, and 24% pN1^8^. Since results of randomized ACOSOG-Z0011 trial, cALND is less frequently performed for patients with primary breast conservation therapy and one or two involved sentinel nodes (SNs) by micro- or macro-metastases without extracapsular extension with whole breast radiotherapy and systemic adjuvant treatment (endocrine therapy and or chemotherapy)^9^. Results of IBCSG 23-01 confirmed the non-inferiority of cALND omission for patients with SN isolated tumor cells (ITC) or micro-metastases^10^. However, some limitations to conclude non-inferiority of cALND omission in comparison with cALND were reported, particularly for patients with SN micro-metastases and total mastectomy, related to the very low number of patients included in this situation in IBCSG 23-01 trial. Moreover, neither trial had included patients with SN macro-metastases and total mastectomy or patients with more than two involved SN by macro-metastases and or extracapsular extension.

Before results with 10-years follow-up of these two randomized trial, we started the SERC randomized trial of cALND versus no cALND for patients with SN involvement, whatever the size of metastasis, and with breast conservative surgery or mastectomy. The aim of this study was to analyze treatment delivered and pathologic results of patients included in SERC trial.

## Results

### Patient accruals and characteristics

**Fig. 1:**
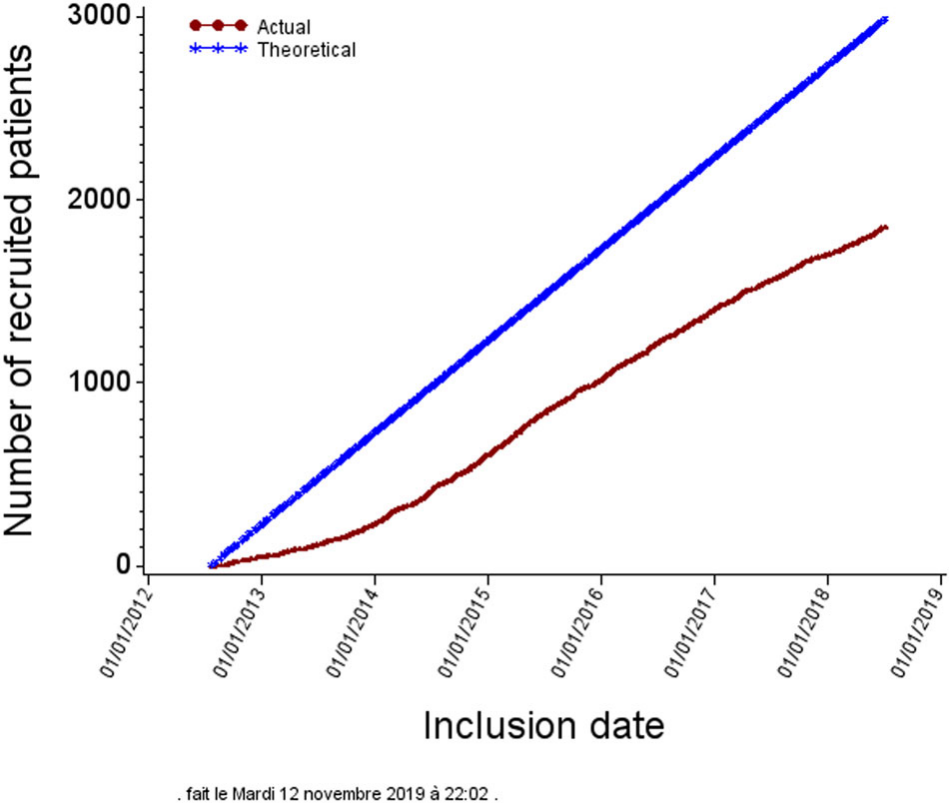
Inclusion of patients. Inclusion number of patients in SERC trial (red line) in comparison with theoretical inclusion number planned (blueline).

**Fig. 2:**
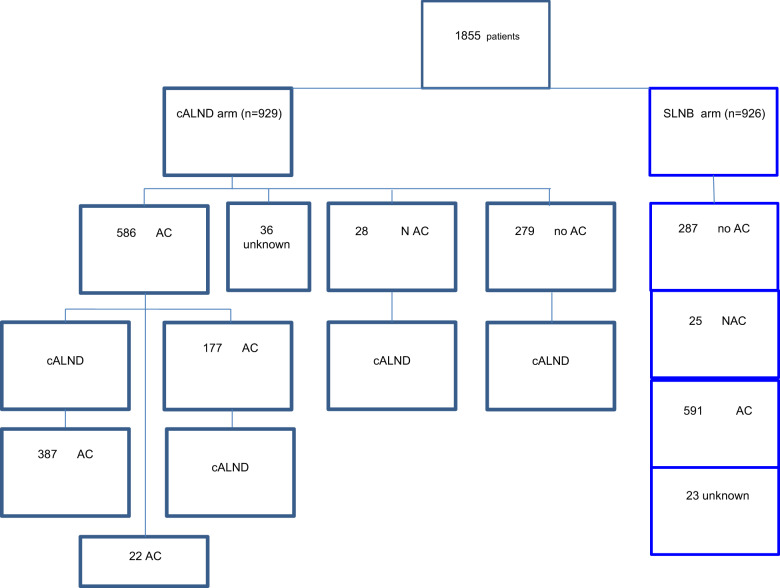
Flow chart. Chemotherapy according to each arms, CALND and SLNB alone. cALND completion axillary lymph node dissection, SLNB sentinel lymph node biopsy, AC adjuvant chemotherapy, NAC neo-adjuvant chemotherapy.

The first 1855 randomized patients were accrued from 53 centers (Fig. 1), of whom 929 were randomized to receive cALND and 926 SLNB alone (Fig. 2).

**Fig. 3:**
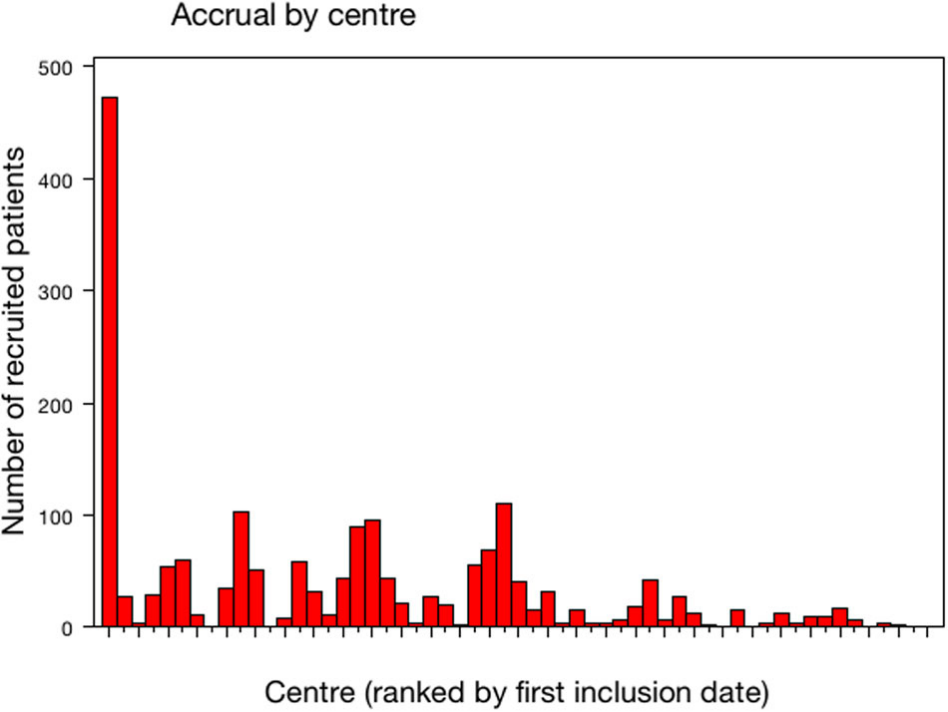
Patient’s number for each center.

All patient demographics and tumor characteristics were balanced between the two randomized arms (Table 1 and Supplementary Data File 1). The number of patients included in each center ranged between 1 and 472 (Fig. 3). Overall, mean age was 57.5 years old (s.d. = 26.0, median = 58, IQR = 49–67) and mean tumor size was 20.3 mm (s.d. = 12.3, median = 18, IQR = 13–25). The median number of harvested SN was 2 (1*n* = 684, 2*n* = 588, >2*n* = 546) and median number of involved SN was 1 (1*n* = 1458, 2*n* = 283, >2*n* = 43: 720 and 738, 151 and 132, 20 and 23 in cALND and no cALND arms, respectively). The status of involved SN was not determined in 180 patients (9.70%). SN ITC was present in 5.91%, micro-metastases in 28.12%, and macro-metastases in 65.97%. Of the 1576 patients with SN micro- or macro-metastases, 583 (37.0%) were non-eligible to Z0011 criteria: extracapsular extension (*n* = 331), mastectomy (*n* = 290), neoadjuvant chemotherapy (NAC) (*n* = 43), >2 involved SN (*n* = 38), and tumor size (*n* = 7). Of the 570 patients with SN ITC or micro-metastases, 18 (3.16%) did not meet the eligibility criteria of IBCSG-23-01: NAC (*n* = 14), >2 involved SN (*n* = 4), and tumor size (*n* = 2). Extracapsular extension was present in 21.5% of patients without difference between two arms (Table 1) with rates of 3.09% (3/97), 7.67% (33/430), and 28.44% (298/1048) for SN ITC, micro-metastases, and macro-metastases, respectively.

**Table 1:** Characteristics of patients according to arms of randomization.

	All		ALND arm		SLNB arm		Chi^2^
	*n*	%	*n*	%	*n*	%	*P*
Randomization	1855		929		926		
**Age**							
≤40	104	5.65	60	6.51	44	4.78	0.256
41–75	1633	88.65	808	87.64	825	89.67	
>75	105	5.7	54	5.86	51	5.54	
Missing data	13		7		6		
**pT size**							
≤10	260	14.32	117	12.90	143	15.73	0.150
10–30	1345	74.06	689	75.96	656	72.17	
>30	211	11.62	101	11.14	110	12.10	
Missing data	39		22		17		
**Grade**							
1	397	22.17	203	22.76	194	21.58	0.205
2	962	53.71	461	51.68	501	55.73	
3	432	24.12	228	25.56	204	22.69	
Missing data	64		37		27		
**LVI**							
No	543	30.99	272	31.12	271	30.87	0.908
Yes	1209	69.01	602	68.88	607	69.13	
Missing data	103		55		48		
**Extracapsular extension**							
No	1340	78.5	680	79.44	660	77.56	0.344
Yes	367	21.5	176	20.56	191	22.44	
Missing data	148		73		75		
**Endocrine receptors**							
Negative	165	9.13	87	9.65	78	8.62	0.449
Positive	1642	90.87	815	90.35	827	91.38	
Missing data	48		27		21		
**Her2**							
Negative	1575	88.58	782	87.77	793	89.40	0.278
Positive	203	11.42	109	12.23	94	10.60	
Missing data	77		38		39		
**Ki67 or MIB1**							
≤10	594	40.74	274	37.95	320	43.48	0.097
11–20	381	26.13	196	27.15	185	25.14	
>20	483	33.13	252	34.9	231	31.39	
Missing data	397		207		190		
**Positive SN number**							
≤2	1741	97.59	871	97.76	870	97.42	0.649
>2	43	2.41	20	2.24	23	2.58	
Missing data	30		16		14		
**SN status**							
pN0 (i+)	99	5.91	45	5.36	54	6.46	0.556
pN1mi	471	28.12	242	28.84	229	27.39	
pN1macro	1105	65.97	552	65.79	553	66.15	
Missing data	180		90		90		
**Chemotherapy**							
No	566	31,51	279	31,24	287	31,79	0,883
NAC	53	2,95	28	3,14	25	2,77	
AC	1177	65,53	586	65,62	591	65,45	
Missing data	59		36		23		
**Surgery**							
Conservative	1466	80.15	744	81.31	722	78.99	0.214
Mastectomy	363	19.85	171	18.69	192	21.01	
Missing data	26		14		12		

*T* tumor, *LVI* lympo-vacular invasion, *SN* sentinel node.

Of the 1855 randomized patients, the actual treatment received was documented in 1823 patients: 840 patients had SLNB followed by ALND, 983 patients had SLNB alone, and the status of ALND was missing in 32 patients. One hundred and eight patients (5.92%) did not receive the study treatment as randomized: 90 in the ALND arm did not have ALND and 18 patients in the SLNB-alone arm had ALND. The main reason for not adhering to the randomized arm was due to the patient’s decision in the ALND arm (83/90), and the investigator’s decision in the SLNB alone arm (11/18),

Overall, these protocol deviations were in relation with patient’s decision in 83 cases (11 for ITC SN, 24 for micro-metastases, 39 for macro-metastases, and 9 with unknown SN status), in relation with investigator’s decision in 18 cases (1 ITC, 4 pN1mi, 12 macro-metastases, and 1 with unknown SN status) and others reasons in 7 cases (1 pN1mi, 4 macro-metastases, and 2 unknown SN status).

In summary, in the ALND arm 12 ITC (12/90: 13.3%), 27 micro-metastases (27/90: 30.0%), 41 macro-metastases (41/90: 45.6%), and 10 unknown SN status did not have ALND and in SLNB arm 2 micro-metastases (2/18: 11.1%), 14 macro-metastases (14/18: 77.8%), 2 unknown SN status have cALND. Consequently, we observed a significant difference between groups according to SN status (*p* = 0.025) with more SN macro-metastases in the ALND group versus SLNB group (67.8% versus 64.4%) (Supplementary Data File 3).

No significant differences in patient’s characteristics (age, grade, tumor histology, tumor size, LVI, HR, and tumor subtypes), type of surgery, and adjuvant chemotherapy (AC) were observed between the two actual treatment groups (Table 1 and Supplementary Data File 3). Total mastectomy was done in 363 patients (19.9%) and SN status rates were 9.7% (31/321) for patients with SN ITC, 25.5% (82/32) for SN micro-metastases, and 64.8% (208/321) for SN macro-metastases. OSNA was use in 4.0% (75/1855) of patients: 3.8% (35/929) and 4.3% (40/926) in arms cALND and no cALND, respectively, 4.2% (35/840) and 4.1% (40/963) in groups of treatment received, cALND and no cALND, respectively.

### Adjuvant treatments

Full treatment information was not available in patients who did not complete the full sequence of treatment at the date of last follow-up for this analysis (*N* = 59).

The status of WBI or PMRT was missing in 80 patients. WBI or PMRT was delivered in 1707 patients (96.2%): 96.4% and 96.1% in ALND group and SLNB group, respectively. PMRT was delivered in 85.7% of patients with mastectomy (305/356): 97.1% (201/207) for macro-metastatic SN, 70.1% (54/77) for micro-metastases, and 35.5% (11/31) for ITC.

The status of AC administration was missing in 59 patients. AC was administered in 65.5% of patients (1177/1796), NAC in 3.0% (53/1796), ET in 90.1%, and the proportions were similar in the two actual treatment groups: 546 of 831 (65.7%) in the ALND group and 630 of 963 (65.4%) in the SLNB alone received AC (28: 3.4% in the ALND group and 25: 2.6% in the SLNB alone received NAC), 654 of 719 (91.0%) in the ALND group and 731 of 829 (89.3%) in the SLNB alone received ET. AC rates were not different in univariate analysis between the two groups ALND and SLNB alone (Table 1). In multivariate analysis, for 1159 patients with ER-positive tumors, AC was significantly more often administered according to age (OR: 6.18, CI 95% 3.17–12.0, *p* < 0.001 and 23.90, 8.04–71.0, *p* < 0.001 for age 41–75 and ≤40 years old, respectively, in comparison with patients >75 years old), presence of lympho-vascular invasion (OR: 0.51, 0.36–0.72, *p* < 0.001), Her2-positive tumors (OR: 21.1, 5.89–75.7, *p* < 0.001), ductal versus lobular invasive carcinoma (OR: 1.91, 1.24–2.94, *p* = 0.003), SN macro-metastases versus ITC (OR: 2.60, 1.31–5.15, *p* = 0.006), WBI or PMRT (OR: 3.93, 1.61–9.59, *p* = 0.003), grade SBR 2 and 3 (OR: 2.83, 2.03–3.95, *p* < 0.001 and 20.94, 11.0–39.9, *p* < 0.0001, respectively), pathologic tumor size 10–30 versus ≤10 mm (OR: 2.02, 1.35–3.00, *p* = 0.005), of borderline significance for pathologic tumor size >30 versus ≤10 mm (OR: 1.79, 0.98–3.25, *p* = 0.057) and >2 involved SN versus ≤2 (OR 3.19, CI 95% 0.86–11.84, *p* = 0.082), without significant difference between ALND and SLNB groups (groups of treatment realized).

For 149 patients with ER-negative tumors, AC was administered in 144 patients (95%: 74 in the ALND group and 75 in the SLNB group) and no AC was delivered in three patients of the ALND group and five patients of the SLNB group.

### Final pathological findings in the ALND group

Of the 840 patients who underwent cALND, the number of involved non-sentinel node (NSN) was reported missing in five patients. The overall rate of positive NSN was 21.1% for patients with cALND (176/835). Crude rates of positive NSN according to SN status were 6.1% for patients with ITC (2/33), 10.3% for SN micro-metastases (22/214), and 25.7% for SN macro-metastases (134/522). Univariate analysis of patient, tumor, and treatment characteristics revealed that positive-NSN rates were significantly correlated with tumor sizes, >2 involved SN, SN status, extracapsular extension, systemic therapy, and radiotherapy (Table 2 and Supplementary Data File 2). In multivariate analysis, for patients treated by radiotherapy, two factors were significantly associated with higher positive-NSN rate: AC administered after cALND versus no AC (OR 2.99, CI 95% 1.70–5.24, *p* < 0.001) and >2 involved SN versus ≤2 (OR 3.45, CI 95% 1.21–9.85, *p* = 0.021). SN macro-metastases versus ITC was found of borderline significance (OR 3.45, CI 95% 1.21–9.85, *p* = 0.084) (Table 3).

**Table 2:** Non-sentinel-node involvement rate.

	ALND		NSN = 0		NSN+		Chi^2^
	*n*	%	*n*	%	*n*	%	*P*
	840		654	78.3	176	21.1	
**Age**							
≤40	48	5.75	41	6.27	7	3.98	0.3241
41–75	739	88.50	578	88.38	156	88.64	
>75	48	5.75	35	5.35	13	7.39	
Missing data	5		5		0		
**pT size**							
≤10	108	12.97	99	15.18	9	5.11	**0.0016**
10–30	633	75.99	485	74.39	143	81.25	
>30	92	11.04	68	10.43	24	13.64	
Missing data	7		7		0		
**Grade**							
1	180	22.0	151	23.63	27	15.52	0.0691
2	429	52.44	326	51.02	100	57.47	
3	209	25.55	162	25.35	47	27.01	
Missing data	22		20		2		
**LVI**							
No	252	31.46	187	29.92	64	37.43	0.0612
Yes	549	68.54	438	70.08	107	62.57	
Missing data	39		34		5		
**Extracapsular extension**							
No	619	78.55	503	81.13	111	68.10	**0.0003**
Yes	169	21.45	117	18.87	52	31.90	
Missing data	52		39		13		
**Endocrine receptors**							
Negative	82	9.90	66	10.19	16	9.14	0.6829
Positive	746	90.10	582	89.81	159	90.86	
Missing data	12		11		1		
**Her2**							
Negative	721	88.25	562	87.95	155	89.08	0.6820
Positive	96	11.75	77	12.05	19	10.92	
Missing data	23		20		3		
**Number SN+**							
≤2	797	97.43	631	98.44	161	93.60	**0.0004**
>2	21	2.57	10	1.56	11	6.40	
Missing data	1		1		0		
**Status SN**							
pN0 (i+)	33	4.27	31	5.07	2	1.27	**<0.0001**
pN1mi	216	27.94	192	31.42	22	13.92	
pN1macro	524	67.79	388	63.50	134	84.81	
Missing data	67		48		18		
**AC**							
No	257		230	35.55	27	15.52	**<0.0001**
before ALND	150		148	22.87	29	16.67	
after ALND	388		269	41.58	118	67.82	
Missing data	90		39		9		
**Surgery**							
Conservative	682	81.29	538	81.76	142	80.68	0.7427
Mastectomy	157	18.71	120	18.24	34	19.32	
Missing data	1		1		0		
**Radiotherapy**							
No	30	3.65	29	4.48	1	0.58	**0.0151**
Yes	793	96.35	618	95.52	172	99.42	
Missing data	17		12		2		

Bold entries indicate statistical significant differences.

**Table 3:** Positive-NSN rates in multivariate analysis for patients with completion ALND and radiotherapy.

	Odds ratio	95% CI	*P* value
**AC**			
No	1		
AC after ALND	**2.99**	1.70–5.24	**<0.0001**
AC before ALND	1.51	0.77–2.98	0.2318
**Tumor size**			
≤10	1		
10–30	1.92	0.86–4.32	0.1124
>30	1.26	0.46–3.43	0.6547
**LVI**			
No	1		
Yes	0.80	0.52–1.23	0.2992
**Grade SBR**			
1	1		
2	1.21	0.66–2.19	0.5376
3	0.89	0.45–1.79	0.7480
Nb positive SN			
≤2	1		
>2	3.45	1.21–9.85	**0.0209**
**Extracapsular extension**			
No	1		
Yes	1.21	0.75–1.94	0.4346
**SN status**			
ITC	1		
Micro-metastases	1.62	0.35–7.59	0.5370
Macro-metastases	3.70	0.84–6.33	0.0842

*LVI* lympo-vacular invasion, *SN* sentinel node, *AC* adjuvant chemotherapy.

Bold entries indicate statistical significant differences.

Crude rates of positive-NSN were 10.5% for patients without systemic therapy (27/257), 17.9% with NAC (5/28), and 26.1% with AC (142/544). Positive-NSN rates were 16.4% for patients with chemotherapy first administrated prior to cALND (29/177) and 30.5% with chemotherapy administered after ALND (118/387). Chemotherapy was administered in 84.5% for patients with involved NSN (147/176). For SN ITC, involved NSN rate was 13.3% (2/15) with chemotherapy administered after cALND and 0/6 for chemotherapy administered before cALND. Involved NSN rates were, respectively, 4.4% (4/92) without chemotherapy, 6.9% (2/29) with chemotherapy administered before cALND, 17.7% (15/85) with chemotherapy administered after cALND for SN micro-metastases, and 14.5% (20/138), 19.3% (19/109), and 35.2% (87/247) for SN macro-metastases, respectively.

We reported only 1 positive NSN in 104 patients (12.5%), 2 positive NSN in 24 (2.9%), and 3 or more in 48 (5.7%), respectively, 6.5% (16/247), 1.6% (4/247), and 1.6% (4/247) for involved SN by ITC or micro-metastases and 14.4% (75/522), 3.3% (17/522), and 8.0% (42/522) for SN macro-metastases. Number of positive-NSN according to administration time of AC were respectively for no chemotherapy, cALND before chemotherapy, and cALND after chemotherapy: only 1 positive NSN in 7.0% (18/257), 12.1% (18/149), and 10.6% (63/387) patients; 2 positive NSN in 2.3% (6/257), 2.7% (4/149), and 3.6% (14/387) patients; and ≥3 positive NSN in 1.2% (3/257), 1.3% (2/149), and 10.6% (41/387) patients (Supplementary Data File 4).

Crude rates of positive NSN for patients with SN macro-metastases were 7.87% (30/381), 7.0% (7/100), and 11.76% (2/17) for patients with 1 or 2 or more than 2 involved SN, respectively (*p* = 0.0411). There was no statistical difference for SN micro-metastases: 4.79% (9/188), 0% (0/17), and 0% (0/1) for patients with 1 or 2 or more than 2 involved SN, respectively (*p* = 0.4327).

Crude rates of positive NSN were 17.6% (74/420) and 26.5% (73/276) for patient’s eligible and non-eligible to Z0011 trial, respectively, 9.8% (23/235) for patients eligible to IBCSG- 23-01 trial (no patient with positive-NSN among 8 patients non-eligible).

Some minor differences of patient’s characteristics between center 1 and others centers is report in Supplemental Data File 5. A very few number of events (*n* = 102) were reported. Consequently higher follow-up is necessary to analyze the non-inferiority of cALND omission.

## Discussion

A total of 1855 patients were included and analyzed in SERC trial. In all, 856 patients from 177 centers were included between May 2001 and December 2014 in ACOSOG-Z0011 trial^9^, 931 patients from 27 centers were included between April 2001 and February 2010 in the IBCSG-23-01 trial^10^, and 233 patients from 18 centers were included between January 2001 and December 2008 in the AATRM trial^11^.

In both Z0011 and IBCSG-23-01 trials under 50% of the initially estimated population in order to demonstrate non-inferiority of omission of cALND were included. SERC trial annually included 314 patients. Z0011, IBCSG-23-01, and AATRM annually included 63,103, and 33 patients.

One hundred and fourteen patients (6.04%) did not receive the allocated treatment. Ninety-five patients randomized in the ALND group did not undergo ALND and 19 patients randomized in the SLND group underwent ALND (2.0%).

These findings are consistent with results of other trials: 43 patients in Z0011 trial did not receive the allocated treatment, 32 in the ALND arm and 11 in the SLND arm; and 31 patients in IBCSG-23-01: with 17 in the ALND group and 14 in the SLND group.

Regarding SN results the following results were found: 1105 (65.97%) macro-metastasis, 471 micro-metastasis (28.12%), and 99 ITCs (5.91%). Nine hundred of our patients were eligible for Z0011 trial: 591 (65.5%) had macro-metastasis versus 430 (50.2%) in Z0011, 311 (34.5%) had micro-metastasis versus 301 (35.2%) in Z0011, and 125 (14.6%) had unknown SN status. In the Z0011 trial there was a significantly different distribution of micro- and macro-metastasis and non-involved SN between both arm: 44.8% micro-metastasis in the no ALND group and 37% in the ALND group, 29 patients had non-involved SN in the no ALND group and 4 in the ALND group. These significant differences render equivalence between both arms difficult to demonstrate. Our population comprised 218 (28.72%) micro-metastasis in the ALND group versus 257 (29.85%) in the SLNB group and 541 macro-metastasis in the ALND group versus 604 in the SLNB group, with no significant difference. When considering patients presenting ITCs or micro-metastatic SNs in our population: 540 (96.77%) were eligible for IBCSG-23-01: 447 (82.78%) micro-metastasis, and 93 (17.22%) ITCs. However SN results between both trials are not comparable as this distribution was not done.

The following immunohistological results were found: there were 169 (9.09%) patients with negative ER and PR, 1691 with positive ER or PR, 82.56% HR+Her2– tumors, 6% triple negative, 8.47% HR+Her2+ and 2.97% HR−Her2+ tumors. Current data from randomized trial do not classify tumors in molecular subtypes. There were 127 (16.4%) patients with negative ER and PR tumors in Z0011, 91 (9.8%) in IBCSG-23-01, and 28 (13.5%) in the AATRM trial^11^.

There were 21.2% patients in our trial who presented with NSN involvement, with 17.62% eligible for Z0011 and 9.79% for IBCSSG-23-01. NSN involvement rates were 27.3, 7.6, and 13% for Z0011, IBCSG-23-01, and AATRM. The difference in terms of NSN involvement rates between our study and Z0011 could be attributed to the higher proportion of patients undergoing ALND after chemotherapy (21.3%: 178/835). Indeed chemotherapy significantly reduces NSN involvement for patients undergoing ALND after chemotherapy and is therefore responsible for downstaging when compared to patients receiving AC or no chemotherapy. The ACOSOG-Z1071 trial^12^ reported comparable findings with a 41% downstaging after NAC. The SENTINA trial reported a 17.8% positive NSN rate for cN0 patients presenting with a negative pre-therapeutic SN^13^. Park et al.^14^ reported a 40.8% downstaging attributed to NAC for patients with positive axillary nodal cytology. These crucial findings can partly explain the very low rate of axillary recurrence in patients for whom cALND was not performed^9,10,15,16^. The following chemotherapy rates were reported: 65.4% in our study, 57.9% in Z0011, 69.4% in IBCSG-23-01, and 92.1% in AATRM.

Tangent radiation fields have an important effect on axillary control with a 10-year axillary recurrence rate of 0.08% for WBI and 0.754% for partial breast radiation (HR 0.25: 0.08–0.75)^13^. In all, 86.3% patients received breast or chest wall radiotherapy, and 89.3%, 89.7%, and 80.6% after breast conservative surgery in Z0011, AATRM, and IBCSG-23-01 trails. Within a very large population of 14,095 patients we observed a very low (0.51%) rate of axillary recurrence. The following factors were significantly associated with a higher risk of axillary recurrence regardless of size of nodal involvement with no difference between patients with or without ALND: high SBR grade tumors, HR-negative, or HER2-positive tumors^16^.

Another factor that should be taken into account is the administration of ET, which can have a therapeutic impact on axillary lymph nodes:^17^ 46.5% (398/856) patients received ET in Z11, 87.8% (817/931) in IBCSG-23-01, 61.6% (133/216) in AATRM, and 90.1% (673/751) in our study.

Eighty-six patients underwent total mastectomy in the in IBCSG-23-01 trial, 18 in the AATRM trial, and 381 in our study. There is currently no information from previous randomized trials on mastectomy with SN macro-metastasis. Hopefully results from SERC, BOOG 2013-07 (ref. ^18^), and SENOMAC trials^19^ should provide sufficient evidence to support omission of in case of SN macro-metastasis after mastectomy. Unfortunately due to insufficient inclusion the BOOG trial was closed.

In all, 27.7% patients (522) included in this study were not eligible for Z11; this population had an increased rate of NSN involvement particularly when cALND was performed prior to chemotherapy. These patients were not included in previous randomized trails and received adjuvant treatments more frequently.

As a result of lacking evidence to support omitting cALND due to underpowered previous trials^20^, there are currently several ongoing non-inferiority randomized trials investigating the possibility of avoiding cALND in case SN macro-metastatic involvement^21–23^. However, recent contributions are in line with the tendency to avoid cALND according to the Z0011 trial’s criteria^24,25^. The AMAROS trial concluded that there was no significant difference in terms of local control between ALND and axillary dissection for patients with SN involvement; however, considering the sample size the authors could not demonstrate equivalence of both techniques^26^. Due to insufficient evidence to support the omission of ALND in case of SN micro-metastasis after a mastectomy, SERC trial evaluated macro-metastatic as well as micro-metastasis and ITCs with a planned stratification based on metastasis size^27^. Moreover, we have recently confirmed the external validity of SERC trial population in comparison with others populations studies for BC patients with SN micro-metastasis^28^.

In conclusion, we reported involved SNs by ITC, micro-metastases, and macro-metastases for early BC in 5.91%, 28.12%, and 65.97%, respectively, without significant difference between two arms for all criteria but with a significant difference between the two groups of patients according to treatment realized. AC administration rates, post-operative radiotherapy rates, and ET rates were not different between the two arms. Higher positive NSN rate was observed for patients with AC performed after ALND (17.65% for SN micro-metastases and 35.22% for SN macro-metastases). In contrast, when AC was administered before ALND, NSN involvement rates were low: 5.71% for SN micro-metastases and 20.31% for SN macro-metastases. These results explain in part the low rate of axillary recurrence for patients without cALND reported in trials and others studies. When cALND is required, AC could be administered before cALND so as not to delay systemic treatment. Of interest, crude rates of positive NSN were higher for patients non-eligible to Z0011 trial (26.45% versus 17.62%).

Due to the evolution of clinical practice since 10-year results of ACOSOG-Z0011 and IBCSG 23-01 trials, an amendment was performed in July 2018 with restriction of inclusions for non-eligible patients to Z11 trial: patients with involved SNs by micro- or macro-metastases and mastectomy, patients with more than two involved SN or extracapsular extension, and patients with SLNB performed before NAC with SN involvement whatever the size of SN involvement. Moreover, results according to tumors subtypes, for non-eligible patients to Z0011 trial who had the higher positive NSN rate and particularly for mastectomy, could potentially lead to practice changes.

## Methods

### Study design

SERC trial (SERC: “Sentinel Envahi et Randomisation du Curage”) is a multicenter prospective randomized non-inferiority phase-3 trial comparing no cALND with cALND in patients with BC and metastasis in the SN, with a stratification planned between SN disease burden (macro-metastases versus ITC or micro-metastases).

This study is registered with ClinicalTrials.gov, number NCT01717131, June 06, 2013.

Ethics approval were obtained from the Institutional Review Board of Paoli Calmettes Institute (SERC-IPC 2012-001) and the National Ethics Committee (2012-A00379-34) and a written informed consent was obtained from all subjects.

The primary objective is to demonstrate that the hazard ratio (SLNB versus cALND) for disease-free survival is significantly less than the non-inferiority margin set to 1.25.

Patients randomized were recruited from 53 institutions over an accrual period of 73 months from July 2012 to July 2018. Since August 2018, we have proposed to continue inclusions in this trial, only for patients who do not meet Z0011 trial criteria. Women eligible for registration could be any age ≥18 years, provided they had no previous or concomitant malignancy, pure ductal carcinoma in situ, previous systemic therapy before SLNB, distant metastases, palpable axillary nodes.

### Patients

Patients with one or more positive SN, multi-centric tumors, ≤cT2 cN0, ITC, or micro-metastases or macro-metastases with or without extracapsular extension, mastectomy or breast conservative surgery, NAC with SLNB before chemotherapy were allowed to participate. Patients with NAC or hormone therapy before SLNB were excluded.

Patients underwent whole breast radiation (WBR) with a sequential boost to the tumor bed in case of breast conservative surgery or post mastectomy radiotherapy (PMRT). Indications for PMRT were based on current guideline in use in each institution (usually all patients with axillary macro-metastasis and patients without axillary macro-metastases but several prognosis factors, i.e., lympho-vascular invasion, young age, pT size >3 or 5 cm). Radiotherapy started 4–8 weeks after surgery or at the end of AC. Indications followed each institution’s guidelines. A total of 50 Gy at ICRU (International Commission on Radiation Units) point were delivered in 25 fractions of 2 Gy over a period of 5 weeks; patients did not receive axillary radiotherapy and the treatment included two tangential fields for both chest wall radiotherapy and WBR. Indications for AC and endocrine therapy were based on current guideline in use in each institution. A permuted block randomization stratified on each participating center and SN disease burden was used.

SN was detected using isotopic detection alone or a combination of colorimetric and isotopic detection after a peri-areolar or tumoral injection.

A preoperative axillary sonography was systematically performed: cN0 patients with suspicious axillary node and axillary positive biopsy could be included. In case of metastatic SN, could be performed, when randomly assigned to the ALND group, either during the initial surgery after an extemporaneous examination of the SN or after definitive examination during a second surgery. For patients with second surgery for cALND, AC was always planned before cALND which was performed before or after AC.

### SN analysis

SNs were sectioned using a 50–200 microinterval between each section and all sections were examined by the pathologist using hematoxylin and eosin staining (HES). In case of negative result using HES, cytokeratin immune staining was employed. A one-step nucleic acid amplification technic was used to examine SNs^29,30^. A CK19 mRNA copy number/μl ranging between 250 and 5000 was defined as micro-metastases and above 5000 as macro-metastases.

### Statistical analysis

The cut-off date for data collection was July 31, 2018. Graphical display of cumulative numbers of accruals since study start and total accruals per participating centers were presented. Patient and tumor characteristics (age, SBR grade, tumor histology, tumor size, lLVI, ER, HER2 status, tumor subtypes), SN biopsy (SN status, SN involvement), and treatment (type of surgery, systemic chemotherapy, endocrine therapy, trastuzumab) were summarized in all randomized patients. Descriptive statistics are presented, both in the ALND and SLNB randomized groups (ALND and SLNB arms) and in the actual ALND and SLNB treatment groups (ALND and SLNB groups), as mean ± standard deviation, median (interquartile range) for continuous data, and frequency (percent) for categorical data. We detailed the deviations from the randomized treatment in each arm. We used univariate analyses and multivariate logistic regression analyses to identify the factors among the patient and tumor characteristics, SN biopsy data, and treatment options significantly associated with AC administration in ER-positive patients and NSN involvement rates in patients receiving radiation therapy. Prior to analysis, individual data were first categorized using predefined thresholds. Only factors significant or of borderline significance in univariate analyses were included as exploratory variables in regression analyses. Statistical analyses were carried out using SAS release 9.4 (SAS-Institute, Inc., Cary, NC). The level of statistical significance was set to 0.05, with no adjustment for multiplicity.

### Reporting summary

Further information on research design is available in the Nature Research Reporting Summary linked to this article.

## Supplementary information

Supplementary Information

Reporting Summary

## Data Availability

Due to the nature of this research, participants of this study did not agree for their data to be shared publicly, so supporting data are not available.
