# Supplementary material for: Sentinel node involvement with or without completion axillary lymph node dissection: treatment and pathologic results of randomized SERC trial
Source: NPJ Breast Cancer. 2021 Oct 8;7:133. doi: 10.1038/s41523-021-00336-3 (PMC8501060; doi:10.1038/s41523-021-00336-3)
Supplement: Supplementary file 1 — Supplementary Information [file 41523_2021_336_MOESM1_ESM.pdf]

SERC trial group[32] :

Aubert Agostini[33]
Olivier Audrin[34].,
Marie Bannier[1],
Issam Baalbaky[35]
Jean-Marie Brandone[36],
Max Buttarelli[1],
Victoire Cabaud[37]
Frédéric Caquant[38]
Gilles Carrasset[39]
Jean-Philibert Combier[40]
Jean-Claude Darmon[6],
Thomas Darnaud[41]
Frédéric Dedecker[42]
Elodie Demblocque[43]
Frédéric Grolier[44]
Brice Gurriet[36]
Renaud Haberstich[45]
Sandra Houlard[46],
Sophie Knight[1]
Jean-Marie Ladonne[47]
Eric Lambaudie[1]
Jean-François Le Digabel[48]
Ludvine Loussert[49]
Catherine Marsollier-Ferrer[50]
Marc Martino[1, 36]
Emile Mereb[51],
Thierry Miras[52],
Pierre Opinel[53],
Christiane Pourny[54]
Sandrine Rua[1]
Laura Sabiani[1],
Nicolas Sterkers[6]
Romain Saint-Supery[55],
Pierrick Theret[56],
Charlotte Vaysse[57]

[33]. Hopital Conception, Marseille, France.
[34] Clinique Saint Michel, Toulon, France.
[35]. Hopital de Compiègne, France.
[36]. Hopital Saint Joseph, Marseille, France.
[37]. Hopital de Chambéry, France.
[38] Hopital privé de Villeneuve d'Ascq
[39] Clinique Sainte Clotilde, Saint Denis, La Réunion, France.
[40]. Hotel Dieu du Creusot, France.
[41]. Hopital de Bastia, France.
[42]. Clinique Saint Ame, Lambres les Douai, France.
[43]. Hopital de Beauvais, France.
[44]. Hopital de Troyes, France.

[45.] Hopital privé Pays de Savoie, Annemasse, France.
[46.] Centre Hospitalier Bretagne Sud, Lorient, France.
[47.] Clinique des Ormeaux, Le Havre, France.
[48.] Hopital privé de l'estuaire, Le Havre, France.
[49.] Groupe Hospitalier Saint Vincent, Strasbourg, France.
[50.] Hopital de Nimes, France.
[51.] Hopital de Manchester, Charleville-Mézières, France.
[52.] Centre Hospitalier William Morey, Chalon sur Saone, France.
[53.] Hopital d'Aix en Provence, France.
[54.]Centre Hospitalier de Soissons, France.
[55.] Maison de santé, Talence, France.
[56.] Centre Hospitalier Saint Quentin, France.
[57.] CHU Toulouse, France.

Supplemental data file 1: Characteristics of patients according to arms of randomization.

		All		ALND arm		SLNB arm		Chi2
		n	%	n	%	n	%	p
Randomization		1855		929		926		
pT stage	pT1	1061	58.30	524	57.71	537	58.88	0.871
	pT2	681	37.42	347	38.22	334	36.62	
	pT3	30	1.65	15	1.65	15	1.64	
	pT4	11	0.60	4	0.44	7	0.77	
	pTx	37	2.03	18	1.98	19	2.08	
	Missing data	35		21		14		
T histology	Ductal	1434	78.49	721	78.97	713	78.01	0.436
	Lobular	222	12.15	101	11.06	121	13.24	
	Mixt	54	2.96	30	3.29	24	2.63	
	Others	117	6.40	61	6.68	56	6.13	
	Missing data	28		16		12		
OeR	Negative	170	9.42	89	9.89	81	8.95	0.495
	Positive	1635	90.58	811	90.11	824	91.05	
	Missing data	50		29		21		
PR	Negative	357	19.76	181	20.07	176	19.45	0.741
	Positive	1450	80.24	721	79.93	729	80.55	
	Missing data	48		27		21		
T phenotype	ER+ Her2+	149	8.42	78	8.81	71	8.03	0.659
	ER+ Her2-	1461	82.59	722	81.58	739	83.60	
	ER- Her2+	53	3.00	30	3.39	23	2.60	
	ER- Her2-	106	5.99	55	6.21	51	5.77	
	Missing data	86		44		42		
Extemporaneous SN analysis	No	1196	66.04	589	65.08	607	67.00	0.390
	Yes	615	33.96	316	34.92	299	33.00	
	Missing data	44		24		20		
SN number	0	5	0.27	3	0.33	2	0.22	0.734
	1	684	37.52	352	38.60	332	36.44	
	2	588	32.25	292	32.02	296	32.49	
	>2	546	29.95	265	29.06	281	30.85	
	Missing data	32		17		15		
Endocrine therapy	No	153	9.95	73	9.49	80	10.4	0.551
	Yes	1385	90.05	696	90.51	689	89.60	
	Missing data	317		160		157		
Trastuzumab	No	1073	85.63	527	84.59	546	86.67	0.295
	Yes	180	14.37	96	15.41	84	13.33	
	Missing data	602		306		296		
Radiotherapy	No	68	3.83	37	4.20	31	3.47	0.427
	Yes	1707	96.17	845	95.80	862	96.53	

	Missing data	80		47		33		
cT stage	cT0-cT1	1223	65.93	609	65.55	614	66.31	0.450
	cT2	594	32.02	297	31.97	297	32.07	
	cT3	9	0.49	4	0.43	5	0.54	
	Missing data	29	1.56	19	2.04	10	1.08	

Legend : T : Tumor, LVI : lympo-vacular invasion, OeR : oestrogen receptor, PR : progesterone receptor, SN : sentinel node

Supplemental data file 2: Non-sentinel-node involvement rate.

		ALND		NSN = 0		NSN +		Chi2
		n	%	n	%	n	%	p
		840		654	78.3	176	21.1	
T histology	Ductal	655	78.16	515	78.39	138	78.41	0.3909
	Lobular	95	11.34	70	10.65	23	13.07	
	Mixt	29	3.46	21	3.20	7	3.98	
	Others	59	7.04	51	7.76	8	4.55	
	Missing data	2		2		0		
Progesterone receptors	Negative	170	20.53	129	19.91	41	23.43	0.3073
	Positive	658	79.47	519	80.09	134	76.57	
	Missing data	12		11		1		
Endocrine receptors	Negative	82	9.90	66	10.19	16	9.14	0.6829
	Positive	746	90.10	582	89.81	159	90.86	
	Missing data	12		11		1		
T phenotype	ER+ Her2+	68	8.37	55	8.66	13	7.51	0.9207
	ER+ Her2-	664	81.77	516	81.26	144	83.24	
	ER- Her2+	27	3.33	21	3.31	6	3.47	
	ER- Her2-	53	6.53	43	6.77	10	5.78	
	Missing data	28		24		3		
Number SN	0	3	0.30	2	0.30	1	0.57	0.7598
	1	327	39.02	263	39.97	63	36.00	
	2	270	32.22	209	31.76	60	34.29	
	>2	238	28.40	184	27.96	51	29.14	
	Missing data	2		1		1		
Chemotherapy	No	257	30.93	230	35.11	27	15.52	**<0.0001**
	NAC	28	3.37	23	3.51	5	2.87	
	AC	546	65.70	402	61.37	142	81.61	

	Missing data	9		4		2		
Endocrine therapy	No	65	9.04	55	9.84	10	6.37	0.1812
	Yes	654	90.96	504	90.16	147	93.63	
	Missing data	121		100		19		
Trastuzumab	No	503	85.25	396	85.16	105	85.37	0.9547
	Yes	87	14.75	69	14.84	18	14.63	
	Missing data	250		194		53		

Supplemental data file 3: Characteristics of patients according to groups of treatment realized: ALND and SLND alone.

		**ALND group**		**SLNB group**		**Chi2**<br>p
Randomization		840		983		
age	<=40	48	5.75	53	5.41	0.951
	41-75	739	88.5	870	88.78	
	>75	48	5.75	57	5.82	
	Missing data	5		3		
pT stage	pT1	479	57.5	578	59.04	0.888
	pT2	319	38.3	358	36.57	
	pT3	13	1.56	17	1.74	
	pT4	4	0.48	7	0.72	
	pTx	18	2.16	19	1.94	
	Missing data	7		4		
pT size	<=10	108	12.97	152	15.57	0.186
	10 to 30	633	75.99	706	72.34	
	>30	92	11.04	118	12.09	
	Missing data	7		7		
T histology	Ductal	655	78.16	773	78.8	0.404
	Lobular	95	11.34	125	12.74	
	Mixt	29	3.46	25	2.55	
	Others	59	7.04	58	5.91	
	Missing data	2		2		
Grade	1	180	22.00	217	22.49	0.365
	2	429	52.44	529	54.82	
	3	209	25.55	219	22.69	
	Missing data	22		18		

LVI	No	252	31.46	287	30.43	0.664
	Yes	549	68.54	656	69.57	
	Missing data	39		40		
Extracapsular extension	No	619	78.55	719	78.58	0,99
	Yes	169	21.45	196	21.42	
	Missing data	52		68		
ER	Negative	85	10.28	85	8.76	0.274
	Positive	742	89.72	885	91.24	
	Missing data	13		13		
PR	Negative	170	20.53	186	19.16	0.465
	Positive	658	79.47	785	80.84	
	Missing data	12		12		
Endocrine Receptors	Negative	82	9.9	83	8.55	0.321
	Positive	746	90.1	888	91.45	
	Missing data	12		12		
Her2	Negative	721	88.25	849	88.99	0.623
	Positive	96	11.75	105	11.01	
	Missing data	23		29		
Ki67 or MIB1	<=10	252	38.01	340	43.09	0.145
	11 to 20	181	27.3	199	25.22	
	> 20	230	34.69	250	31.69	
	Missing data	177		194		
T phenotype	ER+ Her2+	68	8.37	79	8.32	0.73
	ER+ Her2-	664	81.77	792	83.37	
	ER- Her2+	27	3.33	26	2.74	
	ER- Her2-	53	6.53	53	5.58	
	Missing data	28		33		
Extemporaneous	No	533	64.22	655	67.53	0.14

SN analysis	Yes	297	35.78	315	32.47	
	Missing data	10		13		
SN number	0	3	0.36	2	0.2	0.493
	1	327	39.02	357	36.47	
	2	270	32.22	314	32.07	
	>2	238	28.4	306	31.26	
	Missing data	2		4		
positive SN number	<=2	797	97.43	939	97.71	0.704
	>2	21	2.57	22	2.29	
	Missing data	1		2		
SN status	pN0 (i+)	33	4.27	66	7.35	**0.0246**
	pN1mi	216	27.94	254	28.29	
	pN1macro	524	67.79	578	64.37	
	Missing data	67		85		
Chemotherapy	No	257	30.93	308	31.98	0.5866
	NAC	28	3.37	25	2.6	
	AC	546	65.7	630	65.42	
	Missing data	9		20		
AC time	before ALND	150	27.88			
	after ALND	388	72.11			
	Missing data	8				
Endocrine therapy	No	65	9.04	88	10.74	0.265
	Yes	654	90.96	731	89.26	
	Missing data	121		164		
Trastuzumab	No	503	85.25	570	85.97	0.717
	Yes	87	14.75	93	14.03	
	Missing data	250		320		
Surgery	Conservative	682	81.29	777	79.2	0.28

Mastectomy	157	18.71	204	20.8
Missing data	1		2	

Legend : T : tumor, LVI : lympo-vacular invasion, SN : sentinel node, AC : adjuvant chemotherapy.

Supplemental data file 4: Number of positive-NSN.

Number of positive-NSN	1 positive-NSN	2 positive-NSN	>2 positive-NSN
	104 (12.5%)	24 (2.9%)	48 (5.7%)
SN ITC or micro-metastases	16/247 (6.5%)	4/247 (1.6%)	4/247 (1.6%)
SN macro-metastases	75/522 (14.4%)	17/522 (3.3%)	42/522 (8.0%)
no AC	18/257 (7%)	6/257 (2.3%)	3/257 (1.2%)
cALND before AC	18/149* (12.1%)	4/149 (2.7%)	2/149 (1.3%)
cALND after AC	63/387° (10.6%)	14/387 (3.6%)	41/387 (10.6%)

* 1 patient with unknown number of positive-NSN
° 1 patient with unknown number of positive-NSN
SN: sentinel node, AC: adjuvant chemotherapy, cALND: axillary lymph node dissection completion.

Supplemental data file 5 : patient's characteristics between center 1 and others centers

		**All**		**Center 1**		**Other Centers**		**Chi2**
		n	%	n	%	n	%	p
Randomization		1855		472	25.44	1383	75.56	
age	<=40	104	5.65	34	7.20	70	5.11	0.052
	41-75	1633	88.65	404	85.59	1229	89.71	
	>75	105	5.7	34	7.20	71	5.18	
	Missing data	13		0		13		
pT size	<=10	260	14.32	66	14.19	194	14.36	<0.001
	10 to 30	1345	74.06	319	68.60	1026	75.94	
	>30	211	11.62	80	17.20	131	9.70	
	Missing data	39		7		32		
Grade	1	397	22.17	111	24.13	286	21.49	0.178
	2	962	53.71	230	50.00	732	55.00	
	3	432	24.12	119	25.87	313	23.52	
	Missing data	64		12		52		
LVI	No	543	30.99	170	37.53	373	28.71	0.001
	Yes	1209	69.01	283	62.47	926	71.29	
	Missing data	103		19		84		
Extra-capsular extension	No	1340	78.5	357	79.51	983	78.14	0.544
	Yes	367	21.5	92	20.49	275	21.86	
	Missing data	148		23		125		
Endocrine Receptors	Negative	165	9.13	50	10.80	115	8.56	0.149
	Positive	1642	90.87	413	89.20	1229	91.44	
	Missing data	48		9		39		
Her2	Negative	1575	88.58	391	84.09	1184	90.18	0.001
	Positive	203	11.42	74	15.91	129	9.82	
	Missing data	77		7		70		
Ki67 or MIB1	<=10	594	40.74	181	40.86	415	40.81	0.292
	11 to 20	381	26.13	105	23.70	276	27.14	
	> 20	483	33.13	157	35.44	326	32.06	
	Missing data	397		29		366		
positive SN number	<=2	1741	97.59	439	96.91	1302	97.82	0.275
	>2	43	2.41	14	3.09	29	2.28	
	Missing data	30		5		25		
SN status	pN0 (i+)	99	5.91	61	13.20	38	3.13	<0.001
	pN1mi	471	28.12	148	32.03	323	26.63	
	pN1macro	1105	65.97	253	54.76	852	70.24	
	Missing data	180		10		170		
Chemotherapy	No	566	31,51	131	28.42	435	32.58	<0.001
	NAC	53	2,95	25	5.42	28	2.10	
	AC	1177	65,53	305	66.16	872	65.32	

	Missing data	59		11		48		
Surgery	Conservative	1466	80.15	342	73.23	1124	82.53	<0.001
	Mastectomy	363	19.85	125	26.77	238	17.47	
	Missing data	26		5		21		

Legend : T : Tumor, LVI : lympo-vacular invasion, SN : sentinel node.
