# Supplementary material for: Sentinel node involvement with or without completion axillary lymph node dissection: treatment and pathologic results of randomized SERC trial
Source: NPJ Breast Cancer. 2021 Oct 8;7:133. doi: 10.1038/s41523-021-00336-3 (PMC8501060; doi:10.1038/s41523-021-00336-3)
Supplement: Supplementary file 2 — Reporting Summary [file 41523_2021_336_MOESM2_ESM.pdf]

nature research

Corresponding author(s): Gilles Houvenaeghel

Last updated by author(s): Aug 2, 2021

Reporting Summary

Nature Research wishes to improve the reproducibility of the work that we publish. This form provides structure for consistency and transparency in reporting. For further information on Nature Research policies, see our Editorial Policies and the Editorial Policy Checklist.

Statistics

For all statistical analyses, confirm that the following items are present in the figure legend, table legend, main text, or Methods section.

n/a	Confirmed	
☐	☒	The exact sample size (*n*) for each experimental group/condition, given as a discrete number and unit of measurement
☐	☒	A statement on whether measurements were taken from distinct samples or whether the same sample was measured repeatedly
☐	☒	The statistical test(s) used AND whether they are one- or two-sided<br>*Only common tests should be described solely by name; describe more complex techniques in the Methods section.*
☐	☒	A description of all covariates tested
☒	☐	A description of any assumptions or corrections, such as tests of normality and adjustment for multiple comparisons
☐	☒	A full description of the statistical parameters including central tendency (e.g. means) or other basic estimates (e.g. regression coefficient) AND variation (e.g. standard deviation) or associated estimates of uncertainty (e.g. confidence intervals)
☒	☐	For null hypothesis testing, the test statistic (e.g. *F*, *t*, *r*) with confidence intervals, effect sizes, degrees of freedom and *P* value noted<br>*Give P values as exact values whenever suitable.*
☒	☐	For Bayesian analysis, information on the choice of priors and Markov chain Monte Carlo settings
☒	☐	For hierarchical and complex designs, identification of the appropriate level for tests and full reporting of outcomes
☒	☐	Estimates of effect sizes (e.g. Cohen's *d*, Pearson's *r*), indicating how they were calculated

Our web collection on statistics for biologists contains articles on many of the points above.

Software and code

Policy information about availability of computer code

Data collection	custom code was used by Institut Paoli Calmettes data manager
Data analysis	Statistical analyses were carried out using SAS release 9.4 (SAS-Institute, Inc., Cary, NC).

For manuscripts utilizing custom algorithms or software that are central to the research but not yet described in published literature, software must be made available to editors and reviewers. We strongly encourage code deposition in a community repository (e.g. GitHub). See the Nature Research guidelines for submitting code & software for further information.

Data

Policy information about availability of data

All manuscripts must include a data availability statement. This statement should provide the following information, where applicable:

- Accession codes, unique identifiers, or web links for publicly available datasets
- A list of figures that have associated raw data
- A description of any restrictions on data availability

any restrictions on data availability

Field-specific reporting

Please select the one below that is the best fit for your research. If you are not sure, read the appropriate sections before making your selection.

☒ Life sciences ☐ Behavioural & social sciences ☐ Ecological, evolutionary & environmental sciences

For a reference copy of the document with all sections, see nature.com/documents/nr-reporting-summary-flat.pdf

Life sciences study design

All studies must disclose on these points even when the disclosure is negative.

Sample size	The primary objective is to demonstrate that the hazard ratio (SLNB vs ALND) for disease free survival is significantly lower than the non-inferiority margin set to 1.25. A total number of 3000 patients with 588 events have been calculated in order to answer with an 85% power and an error risk of 5%
Data exclusions	no data were excuded
Replication	cannot be reproduced
Randomization	randomization: 1/1 with a stratification planned between SN macro-metastases and ITC or micro-metastases
Blinding	No blinded possible

Reporting for specific materials, systems and methods

We require information from authors about some types of materials, experimental systems and methods used in many studies. Here, indicate whether each material, system or method listed is relevant to your study. If you are not sure if a list item applies to your research, read the appropriate section before selecting a response.

Materials & experimental systems

n/a	Involved in the study	
☒	☐	Antibodies
☒	☐	Eukaryotic cell lines
☒	☐	Palaeontology and archaeology
☒	☐	Animals and other organisms
☐	☒	Human research participants
☐	☒	Clinical data
☒	☐	Dual use research of concern

Methods

n/a	Involved in the study	
☒	☐	ChIP-seq
☒	☐	Flow cytometry
☒	☐	MRI-based neuroimaging

Human research participants

Policy information about studies involving human research participants

Population characteristics	Women eligible for registration could be any age >= 18 years, provided they had no previous or concomitant malignancy, pure ductal carcinoma in situ, previous systemic therapy before SLNB, distant metastases, palpable axillary nodes. Patients with one or more positive SN, multi-centric tumors, <=T2 N0, ITC or micro-metastases or macro-metastases with or without extracapsular extension, mastectomy or breast conservative surgery, neoadjuvant chemotherapy (NAC) with SLNB before chemotherapy were allowed to participate.
Recruitment	Patients randomized were recruited from 53 institutions over an accrual period of 73 months from July 2012 to July 2018.
Ethics oversight	This study is registered with ClinicalTrials.gov, number NCT01717131, June 06, 2013. Ethics approval were obtained from the Institutional Review Board of Paoli Calmettes Institute (SERC-IPC 2012-001) and the National Ethics Committee (2012-A00379-34)

Note that full information on the approval of the study protocol must also be provided in the manuscript.

Clinical data

Policy information about clinical studies

All manuscripts should comply with the ICMJE guidelines for publication of clinical research and a completed CONSORT checklist must be included with all submissions.

Clinical trial registration	ClinicalTrials.gov, number NCT01717131
Study protocol	ClinicalTrials.gov
Data collection	Patients randomized were recruited from 53 institutions over an accrual period of 73 months from July 2012 to July 2018.
Outcomes	The aim of this study was to analyse treatment delivered and pathologic results of patients included in SERC trial.
